# Subconjunctival Choristoma: Description and Surgical Management of Two Pediatric Patients

**DOI:** 10.7759/cureus.99225

**Published:** 2025-12-14

**Authors:** Chloe C Yang, Joshua Anderson, Clark Deem, Emily Tam

**Affiliations:** 1 Ophthalmology, Mary Bridge Children's Hospital, Tacoma, USA; 2 Pathology, Multicare Health System, Tacoma, USA; 3 Ophthalmology, University of Washington, Seattle, USA

**Keywords:** choristoma, conjunctival mass, eye mass, pathology, pediatric ophthalmology, strabismus

## Abstract

Choristomas are benign, normal tissues that grow in an abnormal location, with osseous choristomas being the rarest. In this report, we present the diagnosis and treatment of two patients with subconjunctival lesions that extended to the lateral eyelid, causing eyelid distortion. A decision for surgical intervention was made, and pathological analysis was performed, which was most consistent with osseous choristoma. We present the diagnosis, surgical management, and pathological analysis of two of the rarest conditions.

## Introduction

Ocular choristomas are congenital, sporadic lesions composed of normal tissue located in an abnormal site. Choristomas include limbal dermoids, dermolipomas, complex choristomas, and osseous choristomas [1,2]. Choristomas are overall rare, with osseous choristoma being the rarest subtype [1]. An ocular osseous choristoma is a growth of mature, compact bone within the ocular or periocular soft tissue and represents the rarest form of ocular choristoma, accounting for only 1.7% of all epibulbar choristomas [1]. Given the rarity of this tumor, accurate identification is essential for appropriate diagnosis and management [1-4]. Here, we report the diagnosis and surgical treatment of two patients with epibulbar osseous choristomas. Both patients were treated with excision and eyelid reconstruction and achieved complete resolution. Although osseous choristomas are benign, surgical excision remains the definitive treatment. Informed consent was obtained for both cases.

## Case presentation

Case 1

A nine-day-old presented with a mass lesion in the superolateral orbit since birth.

**Figure 1:**
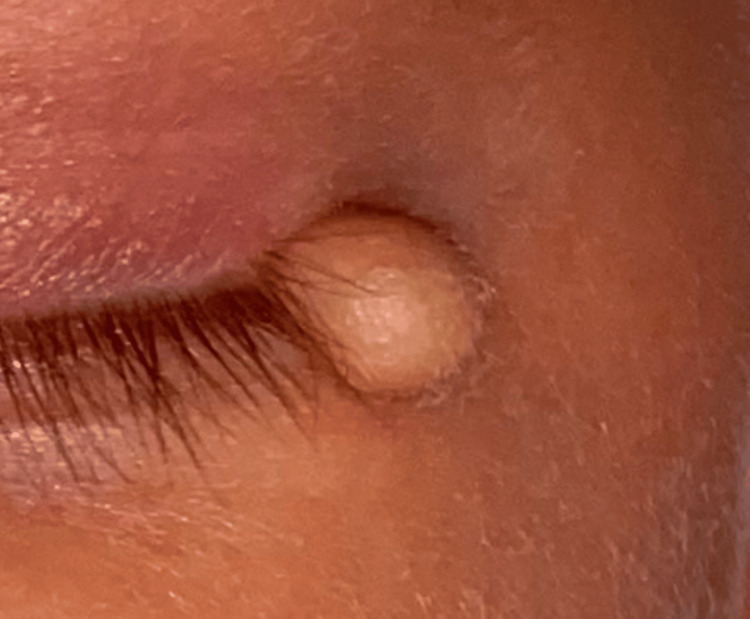
Subconjunctival lesion extending to the lateral canthal region in a nine-day-old newborn

**Figure 2:**
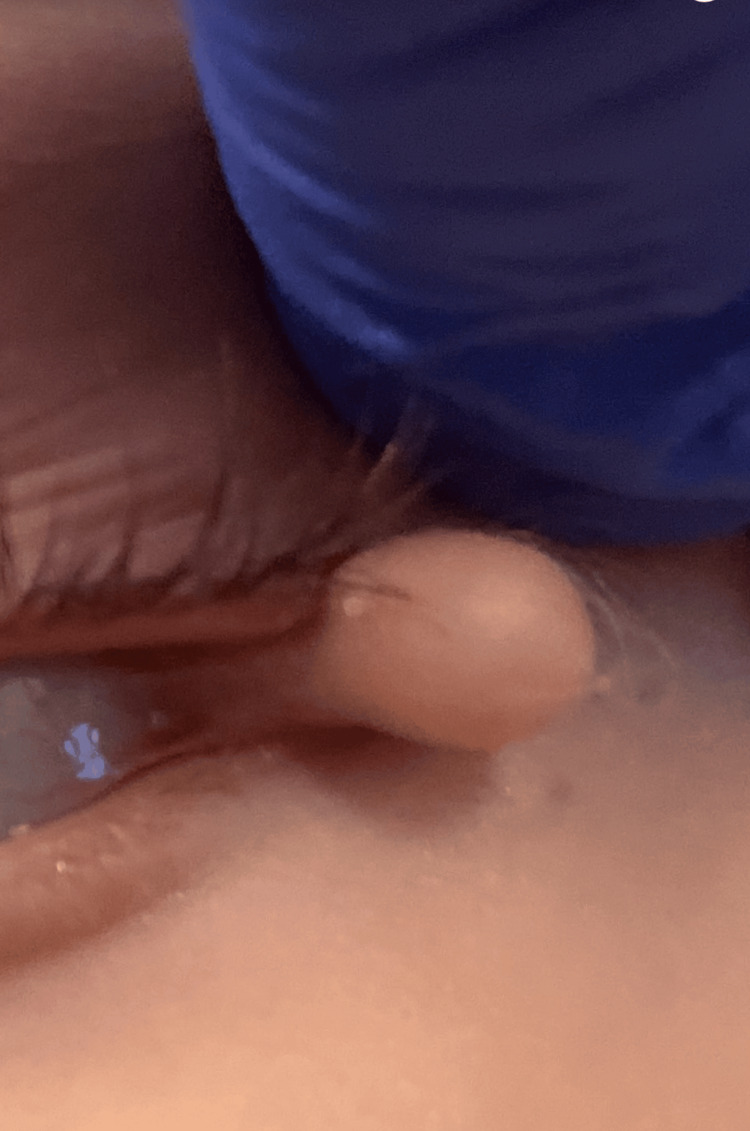
Enlarging subconjunctival mass in a nine-month-old

On examination, the patient appeared healthy, with no dysmorphic facial features or systemic findings. Visual acuity and refraction were appropriate for age and symmetric in each eye, without fixation preference. There was a solitary, ill-defined, mobile subconjunctival mass in the superior temporal fornix (Figure 1). The mass appeared to have hair and was spongy and rubbery, extending from the superior temporal fornix to the lateral canthal region. The lateral canthus was distorted. There was no ulceration, bleeding, eyelash loss, or deep attachment to the orbital rim. Neuroimaging was not performed because the mass was externally located and nonadherent to underlying tissue. Lesion excision was planned because the mass was enlarging over time and caused ocular surface irritation during blinking (Figure 2).

**Figure 3:**
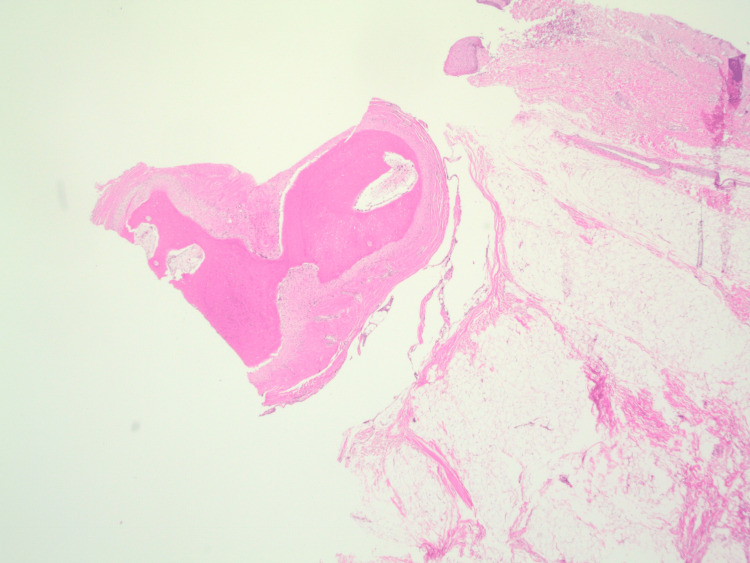
H&E-stained sections from the biopsy showed polypoid skin with abundant subcutaneous adipose tissue. At the deep edge of the biopsy within the subcutaneous tissue, there was a well-circumscribed nodule of dense, mature bone. At high power, the bone showed lamellar growth with surrounding fibrous connective tissue. A few bland rimming osteoblasts were seen. No significant cytologic atypia was identified H&E, hematoxylin and eosin

The procedure was performed under general and local anesthesia. The mass was dissected from the conjunctiva and debulked, with care taken to protect the lacrimal gland and lateral rectus muscle. The mass was excised and sent for biopsy. A linear horizontal incision was made in the lateral canthal region. A 5-0 Vicryl suture was used to reapproximate the upper and lower eyelids to recreate the lateral canthal region. A 6-0 plain gut suture was used to close the skin. Biopsy demonstrated polypoid skin with adipose tissue and mature bone (Figure 3).

Case 2

**Figure 4:**
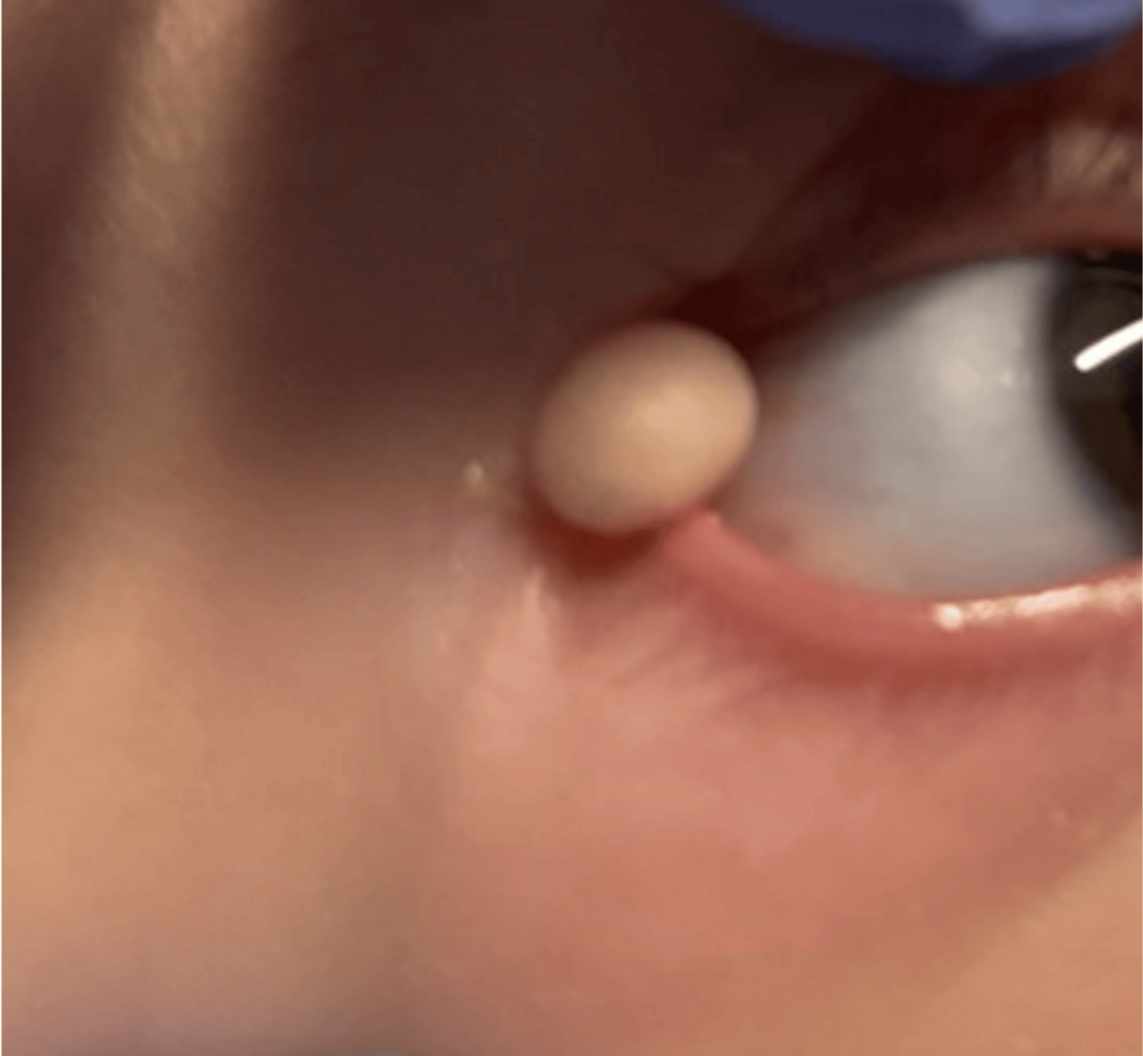
Subconjunctival mass in a two-month-old male

A two-month-old male presented with a mass lesion of the right eye. The examination was nearly identical to that of the first case. The patient appeared healthy, with no dysmorphic facial features or systemic findings. Visual acuity and refraction were appropriate for age and symmetric in each eye, without fixation preference or induced astigmatism. The mass was mobile, subconjunctival, and located in the superior temporal fornix, with attachment to the lateral canthal region (Figure 4). The lateral canthus was similarly distorted to Case 1. There was no ulceration, bleeding, eyelash loss, or deep attachment to the orbital rim. Neuroimaging was not performed given the external location of the mass and lack of adherence to underlying tissue. Over time, the mass enlarged and demonstrated abnormal hair growth. Lesion excision was planned because of progressive growth causing difficulty with complete eyelid closure.

**Figure 5:**
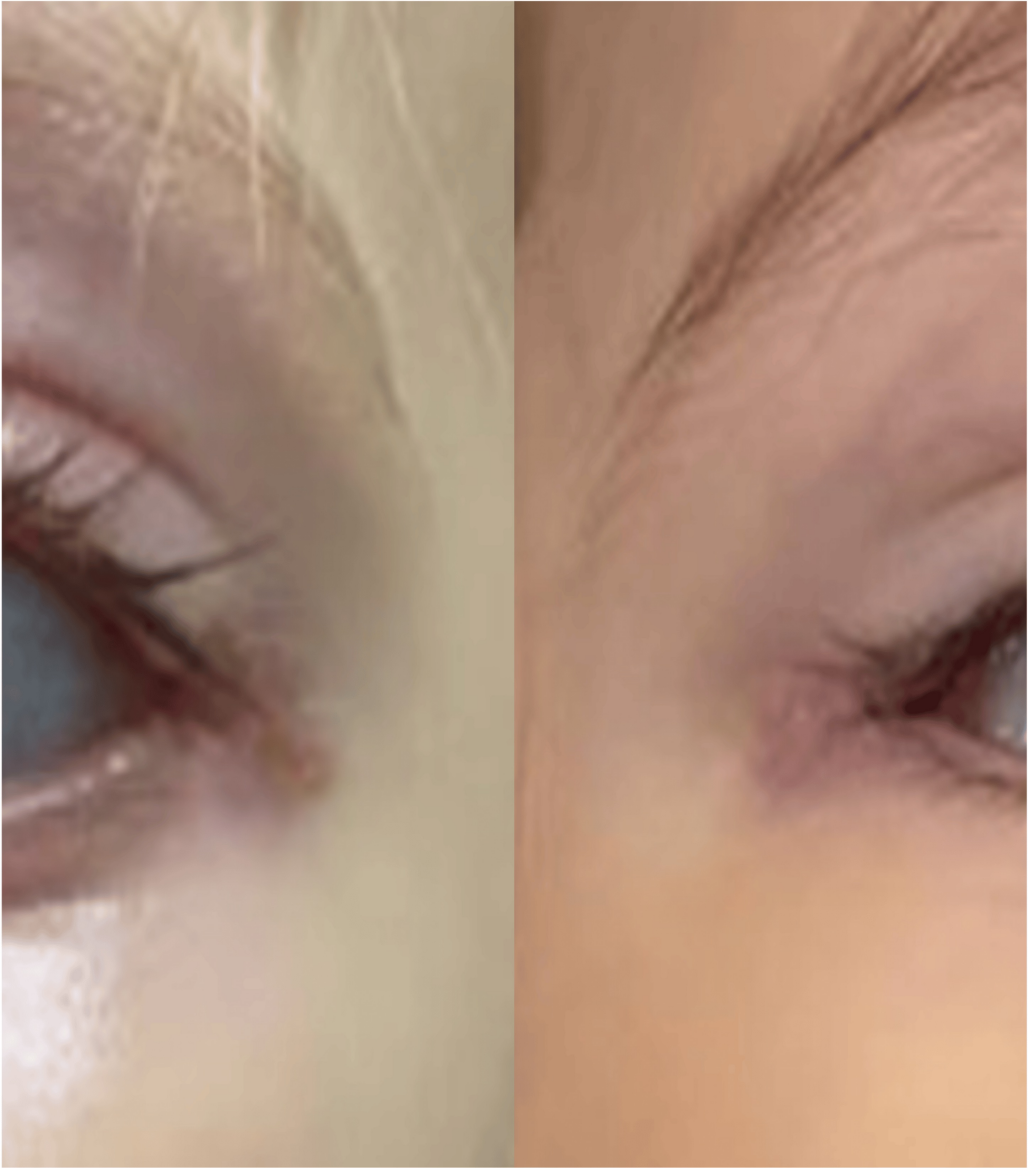
Postoperative week one photographs for Case 1 (left) and Case 2 (right)

**Figure 6:**
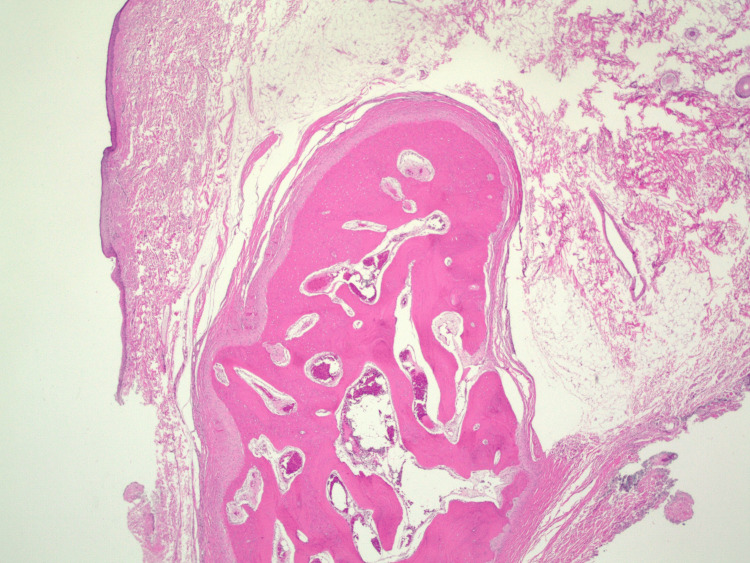
Polypoid skin and subcutaneous tissue containing a well-circumscribed nodule of dense, mature bone within the subcutaneous tissue

The procedure was identical to Case 1. There were no deep scleral attachments. The mass was removed in its entirety and sent for pathologic analysis (Figure 5). Biopsy again showed polypoid skin with adipose tissue and mature bone (Figure 6).

## Discussion

A retrospective cohort study by Aldossary et al. evaluated epibulbar choristoma cases from 2000 to 2016 and found a similar distribution between male and female patients among 120 cases [2]. This is consistent with our cases, which included one female and one male patient. The study also found that subconjunctival masses were commonly located temporally, with 73.3% associated with other ophthalmic manifestations such as eyelid anomalies [2], similar to our cases. In the Aldossary et al. study, 9/120 cases were associated with systemic conditions such as Goldenhar syndrome [2]. Our patients did not undergo genetic testing; however, they did not display any typical features of Goldenhar syndrome.

Histopathologic examination of the choristomas showed polypoid skin and subcutaneous tissue containing a well-circumscribed nodule of dense mature bone. No prominent osteoblastic rimming or cytologic atypia was observed. These findings are similar to those reported by Najmi et al. in a 20-month-old girl from Saudi Arabia [5]. The histopathologic features in that report resemble those seen in our patients and are consistent with osseous choristoma [5]. Although the lesion described by Najmi et al. was similar in appearance and histopathology, that patient had only lateral canthal involvement without episcleral attachment [5].

The differential diagnosis of a pediatric conjunctival mass includes limbal dermoid, melanoma, melanocytosis, myxoma, Kaposi sarcoma, sebaceous carcinoma, extraocular extension of retinoblastoma, intraorbital foreign body, orbital lymphoma, lacrimal gland cyst, orbital fat prolapse, and dermolipoma [6-8]. Shields et al. reported pediatric patients with conjunctival masses referred to an oncology service and found that 67% were melanocytic, 10% were choristomatous, 9% were vascular, and 2% were benign epithelial lesions [6].

Medical management

Dry eye prevention remains a key component in the management of secondary effects of such subconjunctival lesions. Routine screening is also crucial, as these lesions may induce astigmatism and secondary anisometropic amblyopia. Given reports of possible association with Goldenhar syndrome, appropriate genetic evaluation should be considered [2].

Surgical management

The main curative therapy is surgical, as no available treatments can result in resolution of such masses. However, the lesion is thought to have no growth potential [6]. Therefore, indications for surgery include symptoms of tearing, foreign body sensation, irritation, recurrent ocular inflammation, and cosmetic concerns [6]. Given involvement of the lateral eyelid, lateral canthal reconstruction is required for both aesthetic purposes and dry eye prevention.

Given the rarity of this condition, the literature has been largely descriptive and focused on pre-surgical findings, with most studies lacking postoperative follow-up [4-7,9]. In this report, both patients were followed by the same pediatric ophthalmologist from diagnosis through postoperative follow-up (Figure 6).

## Conclusions

Osseous choristoma is the rarest type of ocular choristoma and should be considered in the differential diagnosis of pediatric epibulbar tumors. Surgical excision of eyelid choristoma with eyelid reconstruction should be considered in the presence of recurrent dry eye, difficulty with eyelid closure, amblyopia, or cosmetic concerns. Follow-up is indicated to monitor visual development and manage amblyopia.
